# Trends in Prevalence of Thyroid Cancer Over Three Decades: A Retrospective Cohort Study of 17,526 Surgical Patients

**DOI:** 10.1007/s00268-015-3322-z

**Published:** 2015-11-11

**Authors:** Aleksander Konturek, Marcin Barczyński, Małgorzata Stopa, Wojciech Nowak

**Affiliations:** Department of Endocrine Surgery, 3rd Chair of General Surgery, Jagiellonian University Medical College, 37 Prądnicka Street, 31-202 Krakow, Poland

## Abstract

**Introduction:**

Thyroid cancer (TC) incidence has been increasing in recent years. The aim of this study was to investigate our institution-based estimates of operative volumes for TC over the last three decades.

**Materials and methods:**

This was a retrospective cohort study of patients undergoing thyroid surgery at our institution. Patient characteristics were reviewed in three subgroups: Group I (treated in 1981–1986), Group II (treated in 1987–2002), and Group III (treated in 2003–2012).

**Results:**

TC was diagnosed in 1578/17,526 (9.0 %) thyroid operations. Incidence of TC increased from 3.7 % in Group I to 10.4 % in Group III (*p* < 0.001). Incidence of papillary TC increased form 40.6 % in Group I to 81.3 % in Group III (*p* < 0.001). In the latter group, 23.5 % of all papillary TCs were diagnosed in patients with Hashimoto’s disease. Meanwhile, incidence of anaplastic TC decreased from 16.2 % in Group I to 2.1 % in Group III patients (*p* < 0.001). pT1 tumors were diagnosed in 8.1 % Group I and 54.8 % Group III (*p* < 0.001), whereas pT4 tumors were identified in 40.5 % Group I, 2.4 % Group II, and 0.84 % Group III subjects (*p* < 0.001). pT3 tumors were found in 51.6 % Group I, whereas multifocal papillary TCs were found in 15.7 % Group III patients, the latter with a higher prevalence of pN1 stage (*p* < 0.001).

**Conclusions:**

The following trends in surgical volume for TC were identified throughout the study period: a fivefold increase of thyroid operations for TC, a threefold increase in incidence of papillary TC, and an eightfold decrease in incidence of anaplastic TC. It is of interest that a significant increase in incidence of multifocal papillary TC in young female patients with Hashimoto’s disease was found over time.

## Introduction

From the epidemiological viewpoint, thyroid cancer (TC) occupies a distant place on the list of the most common cancers, accounting for only 1 % of all malignancies, but at the same time, it is the most common malignancy (up to 90 %) of the endocrine system and one of infrequent cancers that are more prevalent in female than in male patients. In recent years, the present authors have been observing a clear increase of TC incidence, and its occurrence is associated with both an excess (papillary thyroid cancer—PTC) and deficit (follicular thyroid cancer—FTC) of dietary iodine. According to the International Agency for Research on Cancer (IARC), the TC incidence rate worldwide falls within a wide range; regions with the highest TC rate include the Pacific Islands (Hawaii), Central America, Japan, East Asia (Korea, Hong Kong) as well as Kuwait and Iceland [1–3]. Compared to other countries, Poland represents rather low incidence rates, nevertheless, according to the National Cancer Register (NCR), the total number of malignant thyroid cases in the Polish population has increased 4 times in females and 3 times in males within the past decades. The above tendencies have been maintained for the past 20 years, indicating that in the Polish society, no such significant increase in the incidence rate has been noted in any other location in the body. At present, it is difficult to say whether this phenomenon results from an absolute increase in morbidity associated with changes in lifestyle, progressive development of the industry and environmental effects, e.g., ionizing radiation (the nuclear catastrophe in Chernobyl), or else from an increase in detectability rates following the availability and improvement of diagnostic techniques (USG + FNAB). The problem is highly important from both the medical and socio-economic viewpoint. On the one hand, a long period of development of highly differentiated thyroid cancer, which is the most common and usually associated with a good prognosis, poses a problem in studies on the epidemiology of this tumor. On the other hand, in spite of improvement of the afore-mentioned diagnostic methods, a high number of cases of thyroid cancer (more than 30 %) are diagnosed only in postoperative histopathology, what necessitates a reoperation in the patient and is associated with increased costs of treatment [4–8].

Clinical material originating from a uniform ethnic area combined with experiences of a center that for many years has been involved in surgical treatment of thyroid diseases have made the authors inclined to perform a comparative analysis of the incidence rates, stages, as well as changes and tendencies in clinical presentation of patients operated on due to thyroid cancer in the years 1981–2012.

## Materials and methods

In the period between January 1981 and December 2012, 17,526 patients were operated on for various thyroid diseases in the clinical center represented by the authors. Of this number, 1578 surgical patients with diagnosed thyroid cancer were selected and subsequently subdivided into three groups, depending on the period when operated on. Group I included patients treated in the years 1981–1986 (Group I *n* = 37), Group II in the period 1987–2002, i.e., at various time intervals after the nuclear catastrophe in Chernobyl (Group II *n* = 590), while the third group consisted of subjects treated surgically in the years 2003–2012 (Group III, *n* = 951). All the patients were classified in keeping with the most recent version of thyroid cancer classification TNM according to UICC (ed.VII, 2010) [9] by means of the analysis of data provided in histological records collected in the Institutional Register of Thyroid Surgery, the classification itself being based on the above presented premises. In the analyzed material of patients treated in the last decade (Group III), establishing the final diagnosis was assisted by the use of immunohistochemistry techniques in 158 (16.6 %) patients.

The collected clinical data were entered into spreadsheets and served as the basis for the statistical analysis of the results. The analysis assumed a 5 and 10-year follow-up.

While analyzing the extent of the primary surgical procedure in the presented groups, a simple division was adopted into total and incomplete (partial resection, subtotal resection, Dunhill procedure, lobectomy with isthmectomy) thyroidectomy. In the analyzed period, all the procedures were performed by an experienced surgical team. The presence of cancer cells in the final histology was an indication for a reoperation and resection of the remaining thyroid tissue [10–14].

All the patients qualified for surgical treatment had current results of TSH, fT4, and fT3 determinations, neck ultrasound, chest X-ray. FNAB as a diagnostic method was not routinely employed in the Group I and in some of Group II patients, hence, it was not included in the presented analysis. On the first postoperative day, all the patients were subjected to indirect laryngoscopy in order to assess vocal cord function, defining paresis as the number of nerves at risk of injury. No restoration of normal mobility of vocal cords after 12 months was defined as permanent paresis. Each time, serum calcium levels were also determined (reference level: 2.15–2.55 mmol/L) after 24 h, assuming the diagnosis of hypocalcemia at the total calcium level below 2.0 mmol/L. Adjuvant treatment, based on the recommendations of the Polish Neuroendocrine Tumor Network Group (Polish Guidelines 2010), with radioactive iodine, introduced in 1991, complemented the surgical therapy and was a significant turning point in the strategy of managing differentiated thyroid cancers. All the patients after surgical treatment required l-tyrosine supplementation, the doses ensuring suppression for the following TSH values: 0.1–0.3 mU/L in the low-risk group, and below 0.1 mU/L in the high-risk group. In addition to postoperative determinations of thyroid-stimulating hormone (TSH), the postoperative follow-up also included the levels of thyroglobulin (TG) and anti-TG antibodies, ultrasonography of the neck and scintiscan of the body.

The investigated groups of patients were compared with respect to age, type, and clinical stage of carcinoma according to pTNM. The assessment of the changeability of the investigated properties was presented as arithmetic means and standard deviations (SD). In the statistical analysis, variables with normal distribution were compared using the Student’s *t* test, while for the remaining variables, the *χ*^2^ test was employed and in case of small sample sizes—the Fisher’s exact test.

All the data were collected prospectively and stored in a computer-based institutional register of thyroid surgery, and analyzed retrospectively by a statistician. Statistical analyses were performed with Statistica 10 for Windows (StatSoft, Krakow, Poland).

## Results

The analysis included 1578 (9.0 %) patients (TC) out of the total number of subjects operated on for various thyroid diseases; primary thyroid cancer was diagnosed in 1548 (98.1 %) patients. Thirty patients (1.9 %) with other types of cancer metastasizing to the thyroid gland were excluded from the analysis. While comparing the investigated groups, the authors noted that initially, incomplete thyroidectomies predominated as the method of treating thyroid diseases, gradually giving way to total resection of both thyroid lobes and central compartment lymph nodes (according to the American Thyroid Association classification), in keeping with the Polish Recommendations modified since 2000. The number of patients followed-up for 5 years was (1327) 84.1 %, while the 10-year follow-up included (368) 23.3 % surgical patients.

Over the more than 30 years, a significant increase in the number of surgical procedures performed in thyroid tumors was seen, amounting respectively to 37 (3.7 %) in Group I, 590 (7.9 %) in Group II and 951 (10.4 %) in Group III. The values were statistically significant (*p* < 0.001) and illustrated a strong (fivefold) increase in the number of surgical procedures performed due to thyroid cancer. All the analyzed groups showed varied ratios of male-to-female patients (Group I = 6:31; Group II = 75:515; Group III = 110:841) however, the differences were statistically non-significant. The mean age of the investigated patients was similar (Group I = 52.0 ± 15.96; Group II = 52.3 ± 15.42; Group III = 52.43 ± 12.7), with a clearly visible tendency towards an increasing risk of thyroid cancer in the population of females in the lower age range, below 45 years of life (<45 years.: Group I: 8 = 21.60 % vs. Group II: 254 = 43.1 % and Group III: 407 = 42.8 %; ≥45 years.: Group I: 29 = 78.4 % vs. Group II: 336 = 56.9 % and Group III: 544 = 57.2 %). In all the groups, papillary thyroid cancer predominated (Group I = 15 (40.6 %); Group II = 405 (68.6 %); Group III = 773 (81.9 %). The values were statistically significant (*p* < 0.001). In the last decade, a significant percentage of papillary thyroid cancer (PTC with HT: 130 = 23.5 %) was concomitant with autoimmune thyroid diseases. The analyzed groups significantly differed with respect to the percentage of anaplastic thyroid cancer—6 (16.2 %) in Group I, 46 (7.8 %) in Group II, and 20 (2.1 %) in Group III (*p* < 0.001), denoting a considerable (eightfold) decrease of the number in the analyzed material. A similar decreasing trend was observed in the percentage of follicular thyroid cancer, which was consistently reduced from 9 cases (24.3 %) in Group I to 91 (9.6 %) in Group III [(Group I = 9 (24.3 %); Group II = 89 (15.1 %); Group III = 91 (9.6 %)] (*p* = 0.01). In the analyzed groups, following the primary procedure, the operation was evaluated as macroscopically residual disease (R2) in 12 (34.4 %) Group I patients, 57 (9.7 %) Group II subjects, and in 4 (0.42 %) patients in Group III (*p* < 0.001) (Table 1).

**Table 1:** Clinical and histopathological factors characteristics of patients with thyroid cancer operated on the years 1981–2012

	Group I [*n* (%)] [37 (3.7)]	Group II [*n* (%)] [590 (7.9)]	Group III [*n* (%)] [951 (10.4)]	*p*
Sex ratio (M:F)	6:31	75:515	110:841	NS
Age (years)				NS
Mean (SD)	52.0 ± 15.96	52.3 ± 15.42	52.4 ± 12.71	
<45[*n* (%)]	8 (21.6)	254 (43.1)	407 (42.8)	
≥45 [*n* (%)]	29 (78.4)	336 (56.9)	544 (57.2)	
Extension of resection of thyroid gland R2 [*n* (%)]	12 (34.4 %)	57 (9.7)	4 (0.42)	<0.001
Histopatology [*n* (%)]				
Papillary TC	15 (40.6)	405 (68.6)	773 (81.3)*	<0.001
Follicular TC	9 (24.3)	89 (15.1)	91 (9.6)	<0.001
Hurthl’e cell TC	0	16 (2.7)	14 (1.4)	NS
Medullary TC	1 (2.7)	16 (2.7)	21 (2.2)	NS
Anaplastic	6 (16.2)	46 (7.8)	20 (2.1)	<0.001
Metastases to thyroid	5 (13.5)	12 (2.0)	13 (1.4)	0.035
Another TC				
Lymphoma, Schwannoma, Sarcoma, Planoepithelial TC	1 (2.7)	6 (2.0)	19 (2.0)	NS
Diameter of the largest foci in mm, mean ± SD	40.17 ± 24.9	19.9 ± 27.3	9.5 ± 15.96	<0.001

* 23.5 % PTC with Hashimoto thyroiditis (PTC with HT)

(*p* < 0.001); *NS* non-significant

All the compared groups of patients also differed with respect to the clinical stage of the tumor at surgery. Stage pT1 was observed in 3 (8.1 %) Group I patients, 22 (37.6 %) Group II subjects, and 521 (54.8 %) Group III individuals (*p* < 0.001), while pT4 was seen, respectively in 15 (40.5 %) Group I patients, 14 (2.4 %) in Group II, and 8 (0.84 %) in Group III (*p* < 0.001). No pT2 tumors were seen in Group I, while an increase of such diagnoses was noted 20 years previously in Group II—267 (45.3 %), and the rate observed in Group III was constant and unchangeable within the last decade—264 (27.8 %). pT3 stage tumors also predominated in Group I—19 (51.6 %) patients; while multifocal tumors with a higher tendency to metastasizing to the lymph nodes were more often seen in Group III—149 (15.7 %) patients. A statistically significant difference was noted in case of lymph node metastases, which predominated in Group III (Group I = 5 (13.5 %); Group II = 108 (18.3 %); Group III = 323 (34.0 %) (*p* < 0.001) contrary to M1, which was significantly higher in Group I than in Group III (*p* < 0.01). The mean size of the largest single tumor focus differed in the analyzed material; tumors with larger diameter predominated in Group I (Group I = 40.17 ± 24.9 mm; Group II = 19.9 ± 27.3 mm; Group III = 9.5 ± 15.96 mm) (*p* = 0.01) (Tables 1, 2).

**Table 2:** TNM factors characteristics of patients with thyroid cancer operated on the years 1981–2012

	Group I [*n* (%)] [37 (3.7)]	Group II [*n* (%)] [590 (7.9)]	Group III [*n* (%)] [951 (10.4)]	*p* *p* < 0.001
pT category [*n* (%)]				
pT1	3 (8.1)	222 (37.6)	521 (54.8)	**<0.001**
pT2	0 (0)	267 (45.3)	264 (27.8)	<0.001
pT3	19 (51.6)	87 (14.7)	158 (16.6)	<0.001
pT4 a	15 (40.5)	14 (2.4)	8 (0.8)	**<0.001**
Multifocal lesions [*n* (%)]	1 (2.7)	156 (25.4)	149 (15.7)	<0.001
Capsular infiltration	34 (91.9)	137 (23.3)	87 (9.1)	<0.010
pN1	5 (13.5)	108 (18.3)	323 (34.0)	**<0.001**
M	3 (8.1)	23 (3.9)	11 (1.2)	<0.001
TNM stage				
I	3 (8.1)	247 (41.9)	570 (59.9)	<0.001
II	0 (0)	152 (25.8)	91 (9.6)	<0.001
III	17 (46.0)	176 (29.8)	281 (29.5)	NS
IVa	13 (35.1)	15 (2.5)	9 (1.0)	<0.001
IVb	4 (10.8)	0 (0)	0 (0)	<0.001

(*p* < 0.001); *NS* non-significant

The analyzed groups significantly differed with respect to complications rate; the authors noted a decreased number of vocal cord paresis [(Group I = 11 (14.8 %); Group II = 54 (4.6 %); Group III = 71 (3.70 %)] with a simultaneous increase in the percentage of transient hypoparathyroidism [Group I = 5 (13.5 %); Group II = 143 (24.2 %); Group III = 264 (27.8 %)] (Table 3).

**Table 3:** Complications after thyroidectomy in patients with TC involved in this study

	Group I [*n* (%)] [37 (3.7)]	Group II [*n* (%)] [590 (7.9)]	Group III [*n* (%)] [951 (10.4)]	*p*
Parathyroid found in pathological report, [*n* (%)]	No data	31 (5.3)	68 (7.2)	0.139
Hypoparathyroidism, [*n* (%)] Total	5 (13.5)	143 (24.2)	264 (27.8)	0.065
Transient (<12 month)	4 (10.1)	135 (22.9)	248 (26.1)	0.053
Permanent (>12 month)	1 (2.7)	8 (1.3)	16 (1.7)	0.758
Unilateral RLN injury, [*n*.(%)]^†^				
Total	11 (14.8)	54 (4.6)	71 (3.7)	<0.001
Transient (<12 month)	8 (10.8)	40 (3.4)	52 (2.7)	<0.001
Permanent (>12 month)	3 (4.0)	14 (1.2)	19 (1.0)	0.051

*RLN* recurrent laryngeal nerve

^†^RLN injury was calculated for nerves at risk and not for patients (there were 74 nerves at risk in Group I; 1180 in Group II; 1902 in Group III)

## Discussion

Thyroid cancer is not found among common tumors. Its incidence is rather low; nevertheless, according to the available data, in the last two decades, the overall morbidity in the Polish population has increased more than fourfold in women and threefold in men. The above tendencies have allowed for formulating a conclusion that there is a significant increase in thyroid cancer incidence that is not observed in tumors situated elsewhere [5, 6].

The afore-mentioned trend is also noticeable in the majority of countries worldwide, including countries of Western Europe (excluding Norway and Sweden) and in North America. In the United States, during the last 10 years, an approximately threefold increase of thyroid cancer was observed. [15–19].

In the present material, the percentage of cancers among patients operated on due to various thyroid diseases also profoundly increased, from 3.7 % in the years 1982–1986 to 10.4 % by the end of 2012. Nevertheless, a fivefold increase in the total number of operations for goiter should be also emphasized. The explanation of this trend continues to provoke acute controversies. Do we indeed face an increase in the incidence rate of thyroid cancer? The availability of various diagnostic imaging studies (USG, USG Doppler, computed tomography, magnetic resonance MRI, or PET-CT), and laboratory tests has affected the detectability rate of all types of cancers, also including thyroid cancer. Thyroid ultrasonography performed incidentally while studying blood flow in the cervical vessels may be here a good example of detecting small, palpably non-diagnostic lesions of the thyroid gland. On the one hand, further diagnostic management follows, which includes fine-needle aspiration biopsy and may be at the same time an indication for a surgical procedure, but on the other, it increases the cost of the procedures. From the medical point of view, detecting a lesion that is in its preclinical stage is absolutely justified and even in view of the slow dynamics of lesion development in differentiated thyroid cancers, considering prolonged mean lifespan noted in the majority of societies, this is a sound management strategy. A strong argument in favor of this fact is limiting the number of difficult reoperations and by the same token, possible complications, and the continuously high number of thyroid cancers that are detected incidentally, although the number and accuracy of FNAB procedures have considerably improved [10, 20–23].

But is it only improved detectability rate that builds the foundations for the increased number of thyroid cancers? If it were so, then with an increasing number of detected small foci of thyroid cancer, there should be noted a consistent decrease in the number of high-stage forms and the total number of tumors. Is it really so? Over the past few years, in the present material, a trend is invariably seen towards a decrease in the number of high-stage forms with infiltration of the capsule and the surrounding structures (pT4: 15 (40.5 %) in Group I, 14 (2.4 %) in Group II, and 8 (0.84 %) in Group III (*p* < 0.001)) in favor of small-sized primary tumors (pT1: 3 (8.1 %) in Group I, 22 (37.6 %) in Group II and 521 (54.8 %) in Group III (*p* < 0.001). Also the mean size of the largest primary foci decreased, respectively, from 40.17 ± 24.9 mm in Group I to <1 cm in Group III (Group III = 9.5 ± 15.96 mm) (*p* = 0.01); nevertheless, the tendency towards an increase of the absolute number of thyroid cancers has been maintained. While analyzing the material, the authors have noted that the group of thyroid cancers with a primary focus greater than 2 cm but smaller than 4 cm has been maintained on the same, fairly unchangeable level. If we thus assume that the availability and development of modern diagnostic methods facilitates detection of small lesions, then clinically detectable by palpation tumors constitute an important group that plays a decisive role in the absolute increase of the number of thyroid cancers. Similar observations have been also made by authors from other countries [24–26].

All the above publications address the analysis of thyroid cancer histiotypes and clearly indicate the prevailing character of papillary carcinoma. A similar situation was noted in the present clinical material, which clearly pointed to an increase of the number of this type of highly differentiated thyroid cancers from 40.6 % in the first analyzed group to more than 80 % in Group III (Group I = 15 (40.6 %); Group II = 405 (68.6 %); Group III = 773 (81.9 %). The difference was statistically significant (*p* < 0.001). In turn, follicular carcinoma predominated in Group I (24.3 %), to become stabilized over subsequent years at the level of 10–15 % of all thyroid cancers (Group II = 89 (15.1 %); Group III = 91 (9.6 %)). (*p* = 0.01). The data are similar to findings presented by other authors [27, 28].

Based on the analysis of the present material, one may state that the above observations may result from the following causes: firstly—from the effect of iodine prophylaxis carried out in Poland, which significantly affected the increase in the percentage of papillary carcinoma, with a decrease in the number of new cases of follicular carcinoma, secondly—from the improved detectability in biopsy materials, and thirdly—as the consequence of re-analysis of histological diagnoses based on immunohistochemistry, what allows for differentiating between the follicular variant of papillary cancer and follicular carcinoma by means of e.g., cytokeratin 19 determinations [29]. Attention should be also directed to the clearly visible increase in concomitant occurrence of papillary thyroid carcinoma and thyroid autoimmune diseases, predominantly Hashimoto’s thyroiditis [30–33].

While analyzing other subtypes of thyroid cancer in the present material, the authors noted a statistically significant decrease in the percentage of anaplastic cancer from 16.2 % in Group I to 2.1 % in Group III (6 (16.2 %) in Group I, 46 (7.8 %) in Group II, and 20 (2.1 %) in Group III (*p* < 0.001)). Based on the analysis of our material and the review of literature, development and availability of modern immunohistochemical methods have favorably affected the higher detectability rate of lymphomas and mixed forms of anaplastic thyroid cancer. An important factor in the etiopathogenesis of this cancer subtype is a long-term history of nodular goiter. Undoubtedly, the profound decrease in the number of anaplastic cancer cases results from a higher availability of diagnostics tests and a decidedly higher number of performed surgical procedures [10].

Another statistically significant difference observed in the analyzed groups was lymph node involvement. In the present material, the authors noted a consistent increase of the number of involved lymph nodes from 13.5 % in Group I to 34.0 % in Group III [(Group I = 5 (13.5 %); Group II = 108 (18.3 %); Group III = 323 (34.0 %) (*p* < 0.001)]. The above tendencies were also seen in other countries, e.g., in more than one-half of the investigated Belarus children after the Chernobyl catastrophe and in the studied Italian and French populations [34]. In the present analysis, the higher percentage of the involved lymph nodes in the investigated Group III was undoubtedly affected by the strategy of surgical management consisting in total extracapsular thyroidectomy along with resection of the central compartment lymph nodes in all cases suspected of thyroid cancer based on the material obtained by fine-needle aspiration biopsy. Although preventive lymphadenectomy in papillary thyroid cancer continues to be highly controversial in view of a higher probability of complications manifested as transient hypoparathyroidism, which—according to data from the literature—is clearly on the rise in this group of patients, such a strategy is also supported by numerous solid arguments. Prophylactic lymphadenectomy of compartment VI cervical lymph nodes often allows for correct determination of tumor stage VI and detection of metastases in preclinical stage, affects formulation of indications for further adjuvant therapy and contributes to decreasing the number of reoperations, thus decreasing the risk of permanent damage to the recurrent laryngeal nerves and hypoparathyroidism. Based on the analysis of the present material, one may state that an increase in the number of cases of transient hypoparathyroidism [Group I = 5 (13.5 %); Group II = 143 (24.2 %); Group III = 264 (27.8 %)] was a consequence of extending the scope of the procedure to include lymphadenectomy of the central compartment lymph nodes, but it also affected the reclassification of tumor stage and shifting patients from Group II to Group III [32, 35, 36].

In summary, it may be stated that despite the fact that from the standpoint of epidemiology, thyroid cancers occupy a distant place accounting for a small percentage of all malignancies, nevertheless, within the past 30 years, a dynamic increase in the incidence of this type of cancer has been observed. Slow dynamics of lesion development and favorable prognoses in the group of highly differentiated thyroid cancers maintain the mortality rate on a continuously unchanged level. Nevertheless, on the one hand, the increase in its incidence in younger women should be borne in mind, and on the other—a prolonged mean lifespan in developed countries; these are important factors in planning the strategy of surgical treatment. In the opinion of the present authors, the above epidemiological considerations and constant improvement of diagnostic methods have become an important element that plays a decisive role in selecting therapeutic methods based on specialized centers.
